# Nanostructured Polymer Thin Films Fabricated with Brush-based Layer-by-Layer Self-assembly for Site-selective Construction and Drug release

**DOI:** 10.1038/s41598-018-21493-9

**Published:** 2018-02-20

**Authors:** Kyungtae Park, Daheui Choi, Jinkee Hong

**Affiliations:** School of Chemical Engineering & Materials Science, Chung-Ang University, 84 Heukseok-ro, Dongjak-gu, Seoul 06974 Republic of Korea

## Abstract

Layer-by-Layer (LbL) self-assembly has been investigated for several decades. However, the conventional LbL method has performance problems on the chair-side caused by its cumbersome and time-consuming process. Thus, we investigate a new LbL self-assembly technique for the fast and high efficient preparation process based on the brush. The multilayer films fabricated by simple sequential brushing of polyelectrolyte solutions are compared to the classical dipping method. We characterize the multilayer films by characteristics such as their morphology and thickness, and compare them against those of the classic method by profilometry, atomic force microscopy. We prepare multilayer films with biocompatible polyelectrolytes, chitosan, and alginate incorporated with a hydrophobic drug carrier. For the drug carrier, a poly(ethylene glycol)-block-poly(ε-caprolactone) (PEG-b-PCL) block copolymer is introduced to construct micelles containing dexamethasone, which is a well-known osteogenesis-inducing drug. The hydrogen bonding behavior between adjacent layers and micelles is investigated by Fourier transform infrared spectroscopy. Additionally, we analyze the release profiles, degradation profiles and toxicity of the multilayer films for biomedical applications. From these results, we can identify the brushing LbL method as a reliable and more efficient multilayer film-construction process compared to conventional dipping LbL, especially for practical applications in dental and clinical situations.

## Introduction

Amphiphilic block copolymers, which have hydrophobic and hydrophilic segments with different solubilities in their structure, have been widely studied for their ability to self-assemble into polymeric micellar structures in selective solutions. Amphiphilic block copolymer micelles can be formed with well-defined nano-sized core-shell structures that possess excellent solubilities in selective solutions, high stabilities, and extended blood circulation periods^1–4^. Contributing to biocompatibility and reduced thrombogenicity of PEG resulting from a brisk chain motion that reduces the interfacial free energy to a low level, poly(ethylene glycol)-*block*-poly(ε-caprolactone) (PEG-*b*-PCL) has attracted enormous attention as a hydrophobic drug carrier over the past few decades^1,5–10^. PCL also has biocompatible and biodegradable properties^11^, so has been studied as the hydrophobic core of polymeric micellar structures for the loading of hydrophobic drugs. However, there are still drawbacks to utilizing block copolymer micelles as a hydrophobic drug delivery platform, such as the difficult quantitative control and undefined release profile when used alone. Therefore, there have been attempts to incorporate block copolymer micelles into a film structure, especially nanoscale multilayer thin films, for controlling the loading amount and release profiles of small molecules or some hydrophobic anticancer drugs^12–14^.

On the other hand, the layer-by-layer (LbL) self-assembly technique has been widely studied; it has become a prevalent coating method for biomedical applications in the past few decades until recently because of its simplicity and versatility to form nanoscale structures^15–21^. LbL self-assembly is a fabrication technique based on the sequential adsorption of materials, especially polyelectrolytes or polypeptides, with mutual interactions with counter materials by electrostatic interactions, hydrogen bonding, hydrophobic interactions, or covalent bonding^12,22^, Also, as the major driving forces of LbL self-assembly are founded on intermolecular interactions, it is a very powerful method for adjusting the characteristics of nano-sized structure of the thin films, such as porosity, thickness, roughness, and density, in the desired way by simply controlling the fabrication conditions including pH, salt concentration, or the number of bilayers^23^. Based on this unique advantages, there have been many attempts to incorporate polymeric nano particles or micelles into multilayer films fabricated by LbL assembly for hydrophobic drug delivery systems. Additionally unlimited kinds of materials can be used in film formation. Owing to these many advantages in film construction, LbL-assembled films have been widely investigated for drug delivery systems and biomedical purposes.

With LbL assembly, there are many different routes to fabricate thin films depending on their purpose, including immersive, spraying, spin coating, and electromagnetic methods, accomplished in preceding research^24^. Among these various techniques, immersion is the dominantly studied technology, also referred to as dipping assembly because its mechanism completely depends on thermodynamics. This means a high reproducibility and reliability of the data results from the basic deposition that relies on the thermodynamic equilibria of the polymers. New technologies are therefore generally compared to the dipping assembly method as the standard technique. However, despite the many advantages and wide availability of applicable materials, some challenges still exist. Firstly, LbL assembly has proven to be a markedly simple and innovative fabrication technique, but its implementation is hindered by its time-consuming nature. Constructing a single bilayer film typically takes 10 minutes of dipping in each polyelectrolyte solutions and then 2 minutes, 1 minute and 1 minute of each washing steps, so 28 minutes at least. Hence, it might take about 5 h for the assembly of a 10 bilayer film. Even spraying and spin coating methods, well-known as faster LbL self-assembly techniques, still need significant time for the preparation of multilayer nanofilms. There is also the limitation in making a selective film on a single substrate because most of the conventional techniques for fabricating nanofilms onto planar surfaces governed by the size and shape of the substrate. In other words, it is fairly difficult to combine more than two different films or partially deposited films onto a single substrate. In addition, from the clinical point of view, it is necessary for treatments on the chair-side to be delivered by simple methods. Preceding research has covered site-selective LbL assembly^25,26^ but it is hardly applicable for dental, clinical, or biomedical purposes generally performed on the chair-side by doctors, not in a laboratory.

In this study, we investigated the brushing LbL assembly technique in comparison with the conventional dipping method. By overcoming the limitations of the conventional LbL techniques, the potential for the application of the brushing LbL method for dental, clinical, or biomedical purposes is proved. In the past few decades, polysaccharide biopolymers such as alginate and chitosan have been frequently studied for drug delivery, micro- or nano-sized particles, and tissue engineering, because of their biocompatibility, non-immunogenic responses, and easily modified properties by pH control^27–31^. Therefore, we chose chitosan and alginate as the building blocks of the multilayer film prepared by the brushing LbL technique. Assembly was followed by thickness measurements, atomic force microscopy (AFM), Fourier transform infrared (FT-IR) spectroscopy, quartz crystal microbalance (QCM) measurements, and contact angle analysis to characterize the films and their properties and compare them to those fabricated with the conventional dipping method. In addition, we used a PEG-*b*-PCL block copolymer for constructing micelles to act as hydrophobic drug carriers incorporated with dexamethasone (Dex), which is a well-known osteogenesis-inducing drug^32,33^. To determine the drug delivery properties, we measured the release profiles of the model drug, degradation profile of the multilayer film, and toxicity of the released solution.

## Materials and Methods

### Materials

Poly(acrylic acid) (PAA, M.W. = 1800), alginic acid sodium salt (Alginate, ALG, product number 180947), and chitosan (CHI, medium molecular weight) were purchased from Sigma-Aldrich. Poly(allylamine hydrochloride) (PAH, M.W. = 12000–200000) was purchased from Polysciences Inc., USA. We used fluorescein isothiocyanate isomer I (FITC, M.W. = 389.38,) to prepare a fluorescent conjugated polymer, PAH-FITC. We synthesized FITC-labeled PAH following a previously published procedure^34^. We prepared coumarin 6 (M.W. = 350.43, ≥99%) as a model drug for measuring the release profiles and used dexamethasone (D4902) as a model osteogenesis-inducing drug. FITC, coumarin 6 fluorescence dyes and dexamethasone were purchased from Sigma-Aldrich. Poly(ethylene glycol)-*block*-poly(ε-caprolactone) (PEO_5K_-*b*-PCL_10K_) was obtained from Polysciences Inc., USA, for constructing the PEO-*b*-PCL micelles. Organic solvent, *N*,*N*-dimethylformamide (DMF, D0558), were purchased from Samchun, Korea. We obtained paint brushes (series 948 #4, 11.5 mm width) from HwaHong, Korea. Phosphate buffered saline (PBS) 10X was obtained from Gibco® Life Technologies.

### Synthesis of dexamethasone- and coumarin 6-loaded micelles

A few methods for preparing PEO-*b*-PCL block copolymer micelles have been reported^2,3,35^. We prepared the block copolymer micelles using a modification of a previously published method^35^. The PEO_5K_-*b*-PCL_10K_ block copolymer solution was prepared at a concentration of 5 mg/mL in *N*,*N*-dimethylformamide (DMF). 2 mg of dexamethasone was then dissolved into the block copolymer solution. Using a 100 μL pipet, we transferred the solution into 20 mL of deionized water (DIW), without adjusting the pH, under vigorous stirring at 1500 rpm. As a result, crew-cut type Dex-loaded micelles were assembled with a hydrophobic interior containing the drug. After 1 h to allow stabilization, we used a 0.2 μm pore size syringe filter to remove the aggregated micelles to achieve a uniform size distribution in the solution. Subsequently, the micelle solution was dialyzed for 3 days in DIW to remove the remaining organic solvent. The micelle size was measured by the Dynamic Light Scattering(DLS) machine. (SZ-100, Horiba) For the coumarin 6-loaded micelles, which were prepared as a model drug carrier, we simply added 1 mg of the coumarin 6 into the PEO_5K_-*b*-PCL_10K_ block copolymer solution instead of the dexamethasone.

### Preparation of film substrates and polyelectrolyte solutions

We fabricated the multilayer films on a silicon wafer by the LbL assembly method using PAA, PAH, CHI, ALG, and the Dex-loaded PEO-*b*-PCL block copolymer micelle solutions. For the LbL assembly based on intermolecular electrostatic interactions, silicon wafers and glass substrates were prepared by a 2 min oxygen plasma treatment to become negatively charged with O_2_ plasma treatment machine. (CUTE-1B, Femto science, Korea) The concentrations of the PAA and CHI solutions were 5.0 mg/mL. Both PAH and FITC were added at 10.0 mg/mL to give the PAH-FITC solution. ALG was added at 5.0 mg/mL to the Dex-loaded PEO-*b*-PCL block copolymer micelle solution. The pH values of the polyelectrolyte solutions were adjusted as follows: PAA, pH 4.0; PAH-FITC, pH 8.0; CHI, pH 5.0; ALG + micelle blend, pH 2.5. The pH influences the ionization of amine and carboxyl groups and the surface charge density of the materials used^36^. The pH values of the solutions were controlled using 0.1 M HCl and 0.1 M NaOH solutions and measured via benchtop pH meter (HI 2211 PH/ORP meter, HANNA instruments).

### Preparation of multilayer thin films fabricated by conventional LbL self-assembly and the brushing LbL method

We fabricated multilayer thin films by conventional LbL self-assembly^19,37^ and the brushing LbL method. Firstly, for the conventional LbL method, the treated substrate (silicon wafer or glass) was dipped into the positively charged (PAH, PAH-FITC, or CHI) solution for 10 min. Then, the substrate was washed three times for 2, 1, and 1 min each in DIW. The substrate was then dipped into the negatively charged (PAA, ALG, or ALG + micelle) solution for 10 min and followed by the same washing steps. This cycle produced a single bilayer of positively and negatively charged polyelectrolytes; we repeated this cycle until the intended number of bilayers was deposited.

To prepare thin films by the brushing LbL method, a substrate fixed on a supporting wall was brushed with a polyelectrolyte solution and washed three times with DIW using a disposable pipet. The substrate was dried simply using an air gun before brushing the next polyelectrolyte solution. We repeated these steps to stack the chitosan and alginate + Dex-loaded micelle blend layers until a specific number of bilayers (*n*) was reached to obtain the (CHI/ALG + micelle blend)_*n*_ multilayer thin film. Additionally, for fabricating the multilayer films on the QCM electrode, we used a 50 ml conical tube as the supporting wall and hold the edge of it with tweezers to avoid other unexpected motion.

### Characterization of the multilayer thin films

The thicknesses of the multilayer thin films were measured using a profilometer (Dektak 150, Veeco). The total mass of material deposited in each layer was obtained by a QCM (QCM200, 5 MHz, Stanford Research Systems). The root-mean-square (rms) roughness, *R*_q_, and surface topography were measured with an AFM (NX10, Park Systems). The static contact angles (SCA) were measured under ambient conditions by a contact angle goniometer designed in our laboratory. Five 4 μL water droplets were imaged on each sample with a charge-coupled device (CCD) camera (IMT 3, IMT Solutions) and the free ImageJ software and the LB-ADSA plugin were used for contact angle analysis. FT-IR spectra were investigated to characterize the interaction between ALG and the Dex-loaded block copolymer micelles.

### Drug release profiles of the multilayer thin films

For investigating the release profile of the coumarin 6 model drug, a (CHI/ALG + micelle blend)_50_ multilayer film was immersed in 5 mL PBS (1X) mixed with ethanol (EtOH; PBS/EtOH = 7:3) and incubated at a certain pH and temperature. Ethanol was added because the hydrophobic coumarin 6 was insoluble in PBS. Afterwards, 0.5 mL of the solution was collected at regular intervals and added to 0.5 mL of normal PBS. The fluorescence spectra of the coumarin 6 released into the PBS at each stage was measured by a microplate reader (Synergy H1 Hybrid Multi-Mode Microplate Reader, BioTek, USA). The concentrations of coumarin 6 were obtained from a calibration curve calculated by the fluorescence emission (*λ*_ex_ = 480 nm, *λ*_em_ = 520 nm). Additionally, we measured the decrease in film thickness in PBS and 7:3 PBS/EtOH (pH 7.4) solutions at 37 °C to investigate film stability and the deconstruction of the multilayer.

### Cytotoxicity effect of the nanofilms by an MTT assay

To test the cell viability of the nanofilms by an MTT (3-(4,5-dimethylthiazol-2-yl)-2,5-diphenyltetrazolium bromide) assay, we seeded HeLa cells into a 12-well plate at 1 × 10^5^ cells per well, and cultivated the cells overnight to attach them to the film surface (*n* = 3). The culture medium was removed and replaced with fresh medium and the released solution from the film were replaced. After 1 day incubation, 10% MTT in PBS (5 mg/mL) was incubated with Dulbecco’s Modified Eagle Medium (DMEM) for 2 h. The relative amounts of viable cells were compared by reading the optical density at a wavelength of 540 nm. The growth medium consisted of DMEM (Gibco) with 10% fetal bovine serum (Welgene, Gyeongsan-si, Korea), and 1% penicillin/streptomycin (Gibco).

## Results and Discussion

### Brush-based layer-by-layer deposition sequence

**Figure 1:**
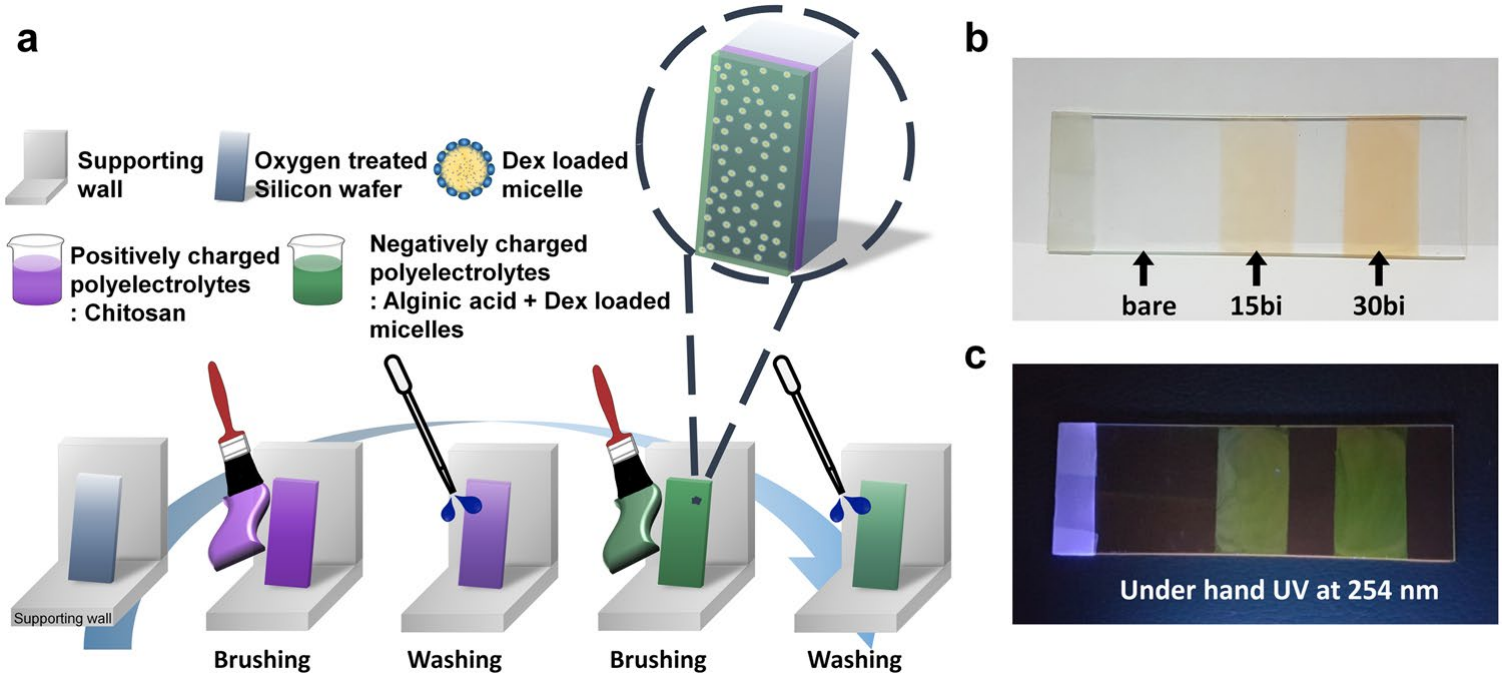
(**a**) Schematic illustration of the brushing layer-by-layer (LbL) self-assembly process on a flat substrate. Photographs of the (PAH-FITC/PAA)_*n*_ (*n* = number of bilayers) multilayer films prepared by brushing LbL on a glass slide under (**b**) visible light and (**c**) ultraviolet (UV) light (*λ* = 254) (The Fig. 1a is representing brushing LbL process and it was drawn by K.P.).

We have introduced a brush-based LbL self-assembly method, described in detail in the schematic illustration in Fig. 1a, where purple represents cations such as chitosan, green represents anions like alginate, and the yellow round particles represent block copolymer micelles containing dexamethasone. For the brushing LbL process, we simply placed the substrates on a wall or other supporting structure oriented vertically on a bench, then we brushed them twice with a polycation solution followed by three rinsing steps with the disposable pipet from top to bottom. Since the substrate surfaces were negatively charged due to the oxygen plasma treatment, we introduced a positively charged solution first. After drying with a blower, we applied a polyanion solution to the substrates by brushing, followed by the rinsing steps. This process resulted in a single bilayer; hence, it was repeated until the desired film thickness was accomplished. Previous research normally used 1 mg/mL polyelectrolyte solutions, which is high enough to construct multilayer films driven by the thermodynamic stabilization of the polyelectrolytes, and the adsorption usually does not depend on the polymer concentration^19,38^. For the brushing LbL method, solution concentrations higher than the general of 5 mg/mL were required, because the film tore off easily after 10 bilayers when using 1 mg/mL of each polycation and polyanion (See Supplementary Fig. S1). The higher concentration gave a uniform film thickness, unlike the 1 mg/mL case from Supplementary Fig. S1a, and viscous properties that made it possible to fabricate films onto the substrate selectively.

We proved the possibility of site-selective deposition via the differences in fluorescence intensity and the depth of color. Figure 1b,c show that the color of the (PAH-FITC/PAA)_*n*_ multilayer film depends on the number of bilayers, *n*. As the number of bilayers increased, the fluorescence intensity under a hand UV-vis was stronger and the color deepened, resulting from the increased amount of FITC-conjugated PAH. Thus, we could fabricate films of different thicknesses onto a single glass substrate in any desired pattern or shape. Also, with relatively short process time and enough washing steps, we could construct uniform structure through the substrate. By applying the basic processes of classic LbL assembly to the brushing LbL method, we could take advantage of the favorable aspects of the conventional method by controlling the structure, thickness, uniformity, and morphology of the film through the interaction of the materials at the molecular level^19,39^.

### Film characterization

**Figure 2:**
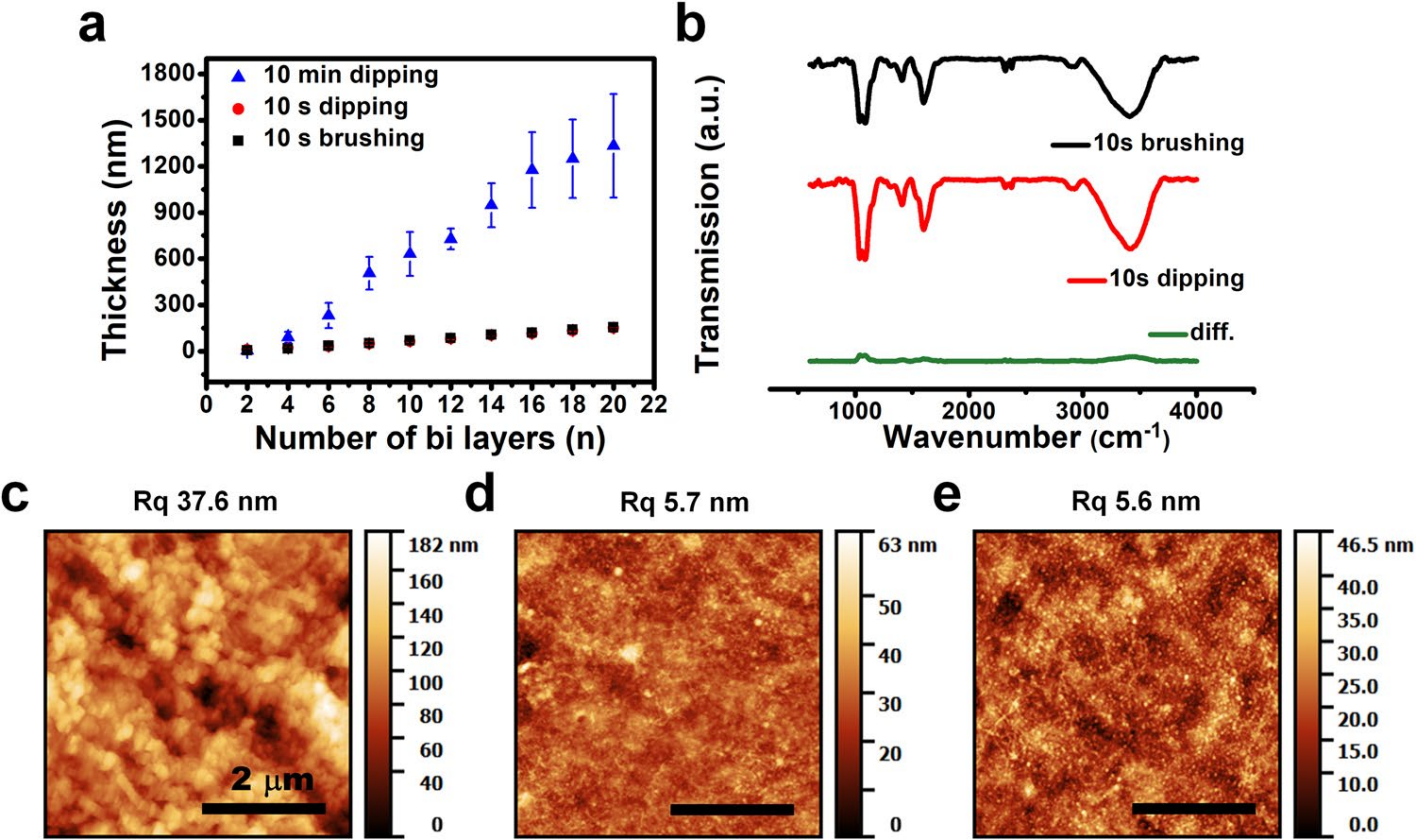
Comparison of the conventional dipping LbL and brushing LbL methods. (**a**) Thickness growth curves of a (CHI/ALG + micelle blend)n multilayer film up to n = 20, fabricated by dipping for 10 min (blue triangles), dipping for 10 s (red circles), and brushing for 10 s (black squares) at two-bilayer intervals. (**b**) FT-IR transmission spectra of the multilayer films fabricated by 10 s brushing LbL (top, black), 10 s dipping LbL (middle, red), and the difference between the two (bottom, green). Topographic images of the multilayer films fabricated with the different methods: (**c**) dipping for 10 min, (**d**) dipping for 10 s, and (**e**) brushing for 10 s.

The LbL assembly of the polyelectrolyte film by the dipping and brushing methods is compared in Fig. 2. The polyelectrolyte solutions used for both deposition methods were the same and the deposition times were standardized to 10 s and 10 min. Figure 2a–e show that dipping and brushing for 10 s lead to the same growth tendencies, and result in more uniform film morphologies than that with the 10 min dipping method (Fig. 2c). The film thickness growth curves show negligible error bars for both 10 s dipping and brushing procedures, and relatively large error bars for 10 min dipping. Furthermore, the rms surface roughness, *R*_q_, of the sample fabricated with 10 min dipping is about seven times greater those of the samples fabricated with the other methods. In addition, the thickness of the 10 min dipping film is almost 10 times thicker (1.3 μm for dipping for 10 min and about 150 nm for 10 s brushing). This significant difference between the two methods is attributed to the inter-diffusion of neighboring layers.

Surface rearrangements take place slowly, while absorbed polyelectrolytes interact with ionized counter polyelectrolytes and attain an equilibrium, or steady-state, over several minutes^40–43^. The brushing LbL technique has a relatively short polymer solution contact time; hence, it has a slight inter-diffusion effect, while the classical dipping method leads to inter-diffusion between neighboring layers and a rough surface morphology.

**Figure 3:**
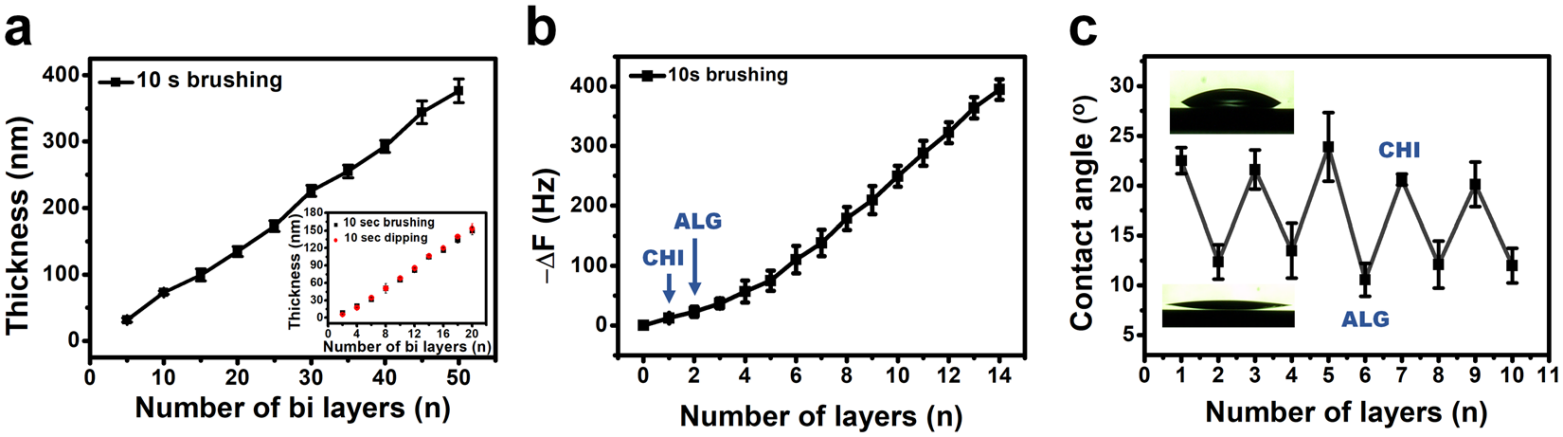
Characterization of the 10 s brushing LbL method by thickness measurement, QCM analysis, and contact angle measurement. (**a**) Thickness growth curves of the (CHI/ALG + micelle blend)_*n*_ multilayer films up to *n* = 50. Inset: Comparison of the dipping and brushing methods for 20 bilayers. (**b**) QCM analysis of the multilayer films up to 14 layers fabricated by the brushing LbL method. (**c**) Static contact angles measurements depending on the alternating outermost layer of the films fabricated by the brushing LbL method. For (**b**) and (**c**), the odd number of layers named as CHI represent chitosan layer and the even number of layers named as ALG represent the ALG + micelle blend outer layers, respectively.

The thickness of the multilayer film prepared by the 10 s brushing LbL procedure indicated linear growth as the number of bilayers increased; the thickness of the 50 bilayer film was 376 ± 18 nm, as shown in Fig. 3a. (The inset of this figure compares the growth curves for the 10 s brushing and 10 s dipping methods for 20 bilayers from Fig. 2a.) The average thickness increment was 7.53 nm per bilayer, which corresponds to the 7.46 nm value obtained from the inset. Both results show the advantages of the brushing LbL method: it can control the film thickness on the nanoscale as well as produce a uniform surface morphology, while the classical dipping method effectuates bulk thickness growth per bilayer.

To demonstrate that the multilayer film prepared by the brushing method also sequentially adhered onto the substrate, i.e., in the layer-by-layer fashion, we performed contact angle and QCM analysis after the deposition of each layer. Figure 3c demonstrates the different contact angles obtained depending on the identity of the outermost layer. The CHI layer and ALG + block copolymer micelle layers were both hydrophilic but with different degrees. For the CHI layer, the SCA was around 22°, and for the ALG + micelle layer, it was approximately half that about 12°. The hydrophilic behavior of both polymer materials is attributed to specific functional groups, such as the hydroxyl and carboxylic groups of ALG and the amine and hydroxyl groups of CHI. Also, the morphology of the multilayer films could affect it. It appeared the polyanion layer composed of ALG and PEG-containing block copolymer micelles has the greater hydrophilicity rather than CHI layer. This might be affected by the well-known hydrophilic characteristic of PEG^44,45^ and rough surface topography of the micelle-containing deposited layer. Thus, we could prove the alternative deposition of the multilayer films via contact angle analysis.

From the QCM data, we could evaluate the sequential layer deposition based on the frequency decrease that is proportional to the increased mass of the deposited layer. In Fig. 3b, odd number of layers represents the CHI layers and the even number of layers represents the ALG + block copolymer micelle layer. From the figure, we could observe the stepwise decrease of the QCM electrode’s frequency as the number of layers increased. For CHI and ALG + block copolymer micelle layers, each material demonstrated a 34.59 ± 6.45 Hz and 36.29 ± 4.08 Hz frequency decrease on average per layer, respectively, after the 6^th^ layer. The exact linear decrease indicates the 10 s brushing method successfully built the film by Layer-by-Layer assembly. And the QCM results from the conventional method with 10 s and 10 min dipping has the same decreasing tendency of the frequency. (See Supplementary Fig. S2) The unexpected peak from 4th layer from the Supplementary Fig. S2b is because of bulk adsorption by the dipping method. From these results, we determined that it is possible to fabricate multilayer films by LbL self-assembly using the brushing method.

### Preparation and characterization of the micelle blend and its incorporation

**Figure 4:**
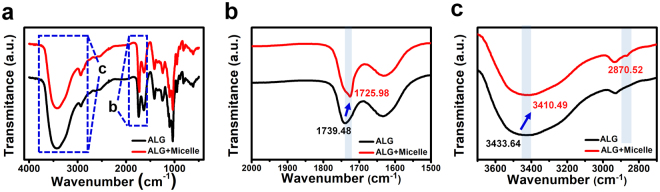
(**a**) FT-IR spectra of the alginate (ALG) solution (black) and the ALG + micelle blended solution (red). Magnified ranges of the FT-IR spectra in (**b**) and (**c**) demonstrate the chemical shifts from the specific bonding.

As we prepared the micelles with PEO_5K_-*b*-PCL_10K_ following the previous method^35^, the micelles could have a crew-cut structure which has a relatively large core than its corona. The PEO part is constituted as corona^46–49^. The average size of the micelles was 118 ± 38.1 nm, measured by dynamic light scattering (DLS) machine. It is a trustworthy result as it compares well with previous data^2,50^. PEO, which forms the corona of the block copolymer micelle, is utilized as a hydrogen bonding acceptor because of its ether group. ALG interacts with PEO as a hydrogen bond donor based on the abundant hydroxyl and carboxyl groups on its structure. For the negatively charged polyelectrolyte solution, we adjusted the pH of the ALG + micelle blend solution to 2.5 because, under the p*K*_a_ of ALG (~3.5–4.4), it has more unionized hydroxyl and carboxyl groups that can act as hydrogen bonding donors. Thus, the hydrogen bonding between the donor hydroxyl and carboxyl groups on ALG and the acceptor parts of the PEO-*b*-PCL micelles made it possible to incorporate these micelles into the film. Figure 4 shows evidence of hydrogen bonding among the ALG and PEO-*b*-PCL micelles. As described in Fig. 4b, the absorption band at 1739.48 cm^−1^ (characteristic of the carboxyl C = O bond) of the ALG solution was shifted to 1725.98 cm^−1^ for the solution blended with the micelles. Also, the maximum absorbance of the peak related to the –OH group was shifted from 3433.64 cm^−1^ to 3410.49 cm^−1^ for the plain and blended solutions, respectively. Both red-shifted peaks were explained by the increased molecular interactions between each molecule based on hydrogen bonding. In addition, the characteristic peaks of PEO-*b*-PCL micelles at 1726.94 cm^−1^ and 2870.52 cm^−1^ (See Supplementary Fig. S3) appeared at Fig. 4c after the ALG and the block copolymer micelles are blended, compared to the pure ALG solution. It proves that the block copolymer micelles were well-incorporated with the ALG.

### Film degradation and drug release

**Figure 5:**
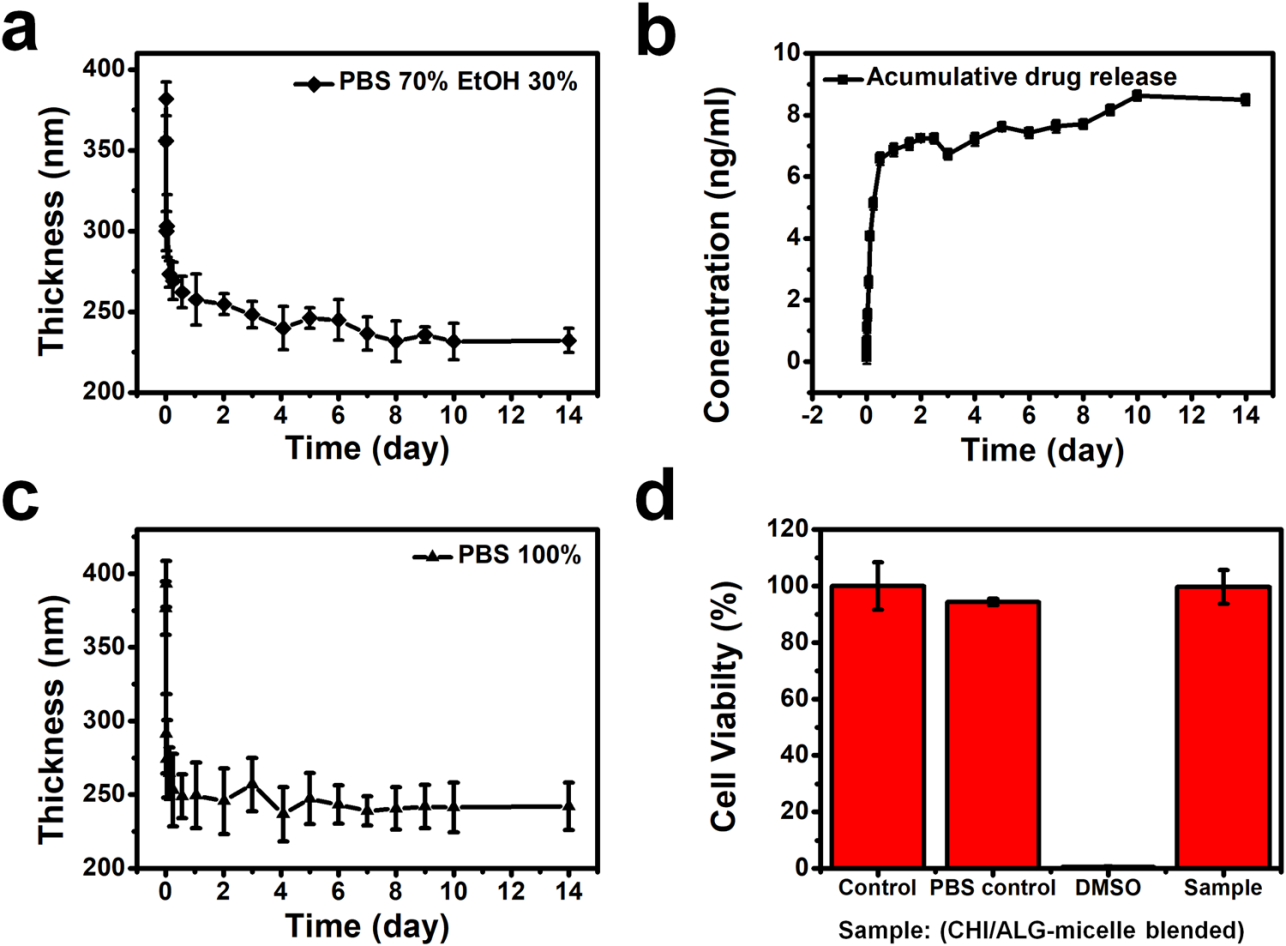
Characteristics of the (CHI/ALG + micelle blend)_50_ multilayer films related to their inherent disassembly behavior. (**a**) Film degradation and (**b**) cumulative release profile of coumarin 6 as a model drug in 7:3 PBS:EtOH (pH 7.4) performed in an incubator at 37 °C. (**c**) Film degradation behavior in 100% PBS (pH 7.4) at 37 °C. (**d**) Cytotoxic effects of the PBS solution of (**c**).

To employ this multilayer film for biomedical purposes, we investigated the film degradation and release profiles, and the cell toxicity of the released solution. Firstly, we measured the fluorescence excitation and emission of the coumarin 6 released from the multilayer film. We simply immersed the multilayer film in 5 mL 7:3 PBS:EtOH solution and took out a small amount of the solution at the indicated time intervals. We introduced ethanol since the coumarin 6, a hydrophobic model drug, was not released from the multilayer film in the 100% PBS solution (See Supplementary Fig. S4). As represented in Fig. 5b, about 80% of the coumarin 6 model drug was released from the multilayer film after 2 days and then more was slowly released until 14 days. The amount of released coumarin 6 was quantified by photoluminescence at an excitation wavelength of *λ*_ex_ = 480 nm and an emission wavelength of *λ*_em_ = 520 nm. The final concentration of the released solution was 8.621 ng/mL, i.e., 21.9 nM, which is above the typical 10 nM dexamethasone concentration of osteogenic media^51^. The molecular weight and structures of coumarin 6 and dexamethasone are similar whilst the latter can slightly dissolve in PBS, so we inferred that more than the typical concentration of dexamethasone required for osteogenic media could be released from the multilayer film.

For analyzing the degradation profile, we measured the thickness of a multilayer film at the same time intervals as used to obtain the release profile. Figure 5a shows the thickness of a (CHI/ALG + micelle blend)_50_ film stored in 7:3 PBS:EtOH solution in an incubator at 37 °C. Figure 5c shows the degradation profile in 100% PBS under the same conditions. Both graphs show a similar tendency of degradation. At pH 7.4, the ratio of protonated amine groups from CHI lessened, so the electrostatic interactions among the building blocks were weakened^52^. Thus, the thickness of each film increased slightly for the first 15 min because of the swelling effect. Then, they decreased gradually until each film reached its final thickness. For Fig. 5a, the thickness of the film immersed in the PBS:EtOH mixed solution decreased from 370 to 240 nm and then saturated, losing 35% of its initial thickness. For the film immersed in the 100% PBS solution, the thickness decreased from 360 to 230 nm, losing 36% of its initial thickness. Considering that chitosan and alginate have very low solubilities in EtOH compared to water, shown in a previous study, a relatively slow film degradation in the 70% PBS and 30% EtOH solution is a reasonable result^53^. In addition, to verify the toxicity of the released solution for further *in vitro* and *in vivo* experiments, a cytotoxic test was carried out. From Fig. 5d, the released sample shows almost the same result as the control, a cell-growth medium. The released sample also showed a slightly higher cell viability than the PBS control, which means the film materials do not have any toxic effect on the cells.

## Conclusion

In this study, we have demonstrated the brushing layer-by-layer assembly method, which is simple and faster compared to the conventional surface coating method. We also demonstrated the possibility of the site-selective deposition of drug release films with different thicknesses. In addition, we have proved that the brush-based fabrication method is based on the principles of layer-by-layer self-assembly by comparing the Atomic force microscopy and Fourier transform infrared spectroscopy data of this method and the classical methods. Static contact angle and Quartz crystal microbalance measurements also supported this finding. For functionalized multilayer films for biomedical applications, we introduced block copolymer micelles incorporated with dexamethasone and investigated the cytotoxicity and release profile. On the basis of this research, the brushing layer-by-layer method represents a new layer-by-layer assembly technique for clinical purposes.

## Electronic supplementary material

Supplementary Info File #1
