# Supplementary material for: Nanostructured Polymer Thin Films Fabricated with Brush-based Layer-by-Layer Self-assembly for Site-selective Construction and Drug release
Source: Sci Rep. 2018 Feb 20;8:3365. doi: 10.1038/s41598-018-21493-9 (PMC5820262; doi:10.1038/s41598-018-21493-9)
Supplement: Supplementary file 1 — Supplementary Info File #1 [file 41598_2018_21493_MOESM1_ESM.pdf]

Supplementary Information

Nanostructured Polymer Thin Films Fabricated with Brush-based Layer-by-Layer Self-assembly for Site-selective Construction and Drug release.

*Kyungtae Park, Daheui Choi and Jinkee Hong**

School of Chemical Engineering and Material Science, Chung-Ang University, 84 Heukseok-ro, Dongjak-gu, Seoul 06974, Republic of Korea.

***Corresponding Autor** : *jkhong@cau.ac.kr*.

Supplementary Figures

Supplementary Figure S1 - Photographic images of the multilayer films fabricated by brush based LbL assembly

Supplementary Figure S2 - QCM analysis of the multilayer film up to 14 layers fabricated by the conventional dipping LbL method

Supplementary Figure S3 - FT-IR spectrum of the dexamethasone-loaded PEO-b-PCL block copolymer micelles dispersed in deionized water without additional pH control

Supplementary Figure S4 - Comparison of the cumulative release profiles of coumarin 6, as a model drug

Supplementary Figure S1

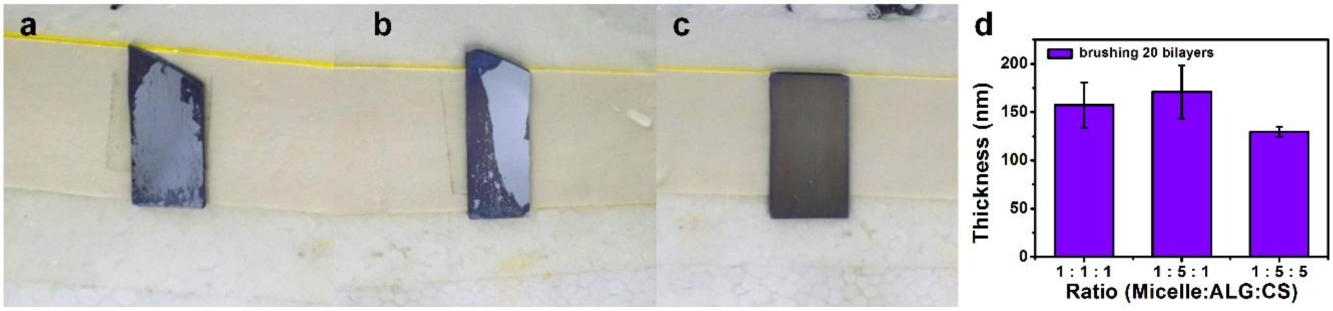

Figure S1 Photographic images of the multilayer films fabricated by brush based LbL assembly onto the silicon wafer substrate with micelle:ALG:CHI ratios of (a) 1:1:1, (b) 1:5:1, and (c) 1:5:5. (d) Thicknesses of the (CHI/ALG micelle + blend)$_{20}$ bilayer films with those concentration ratios for the films.

Supplementary Figure S2

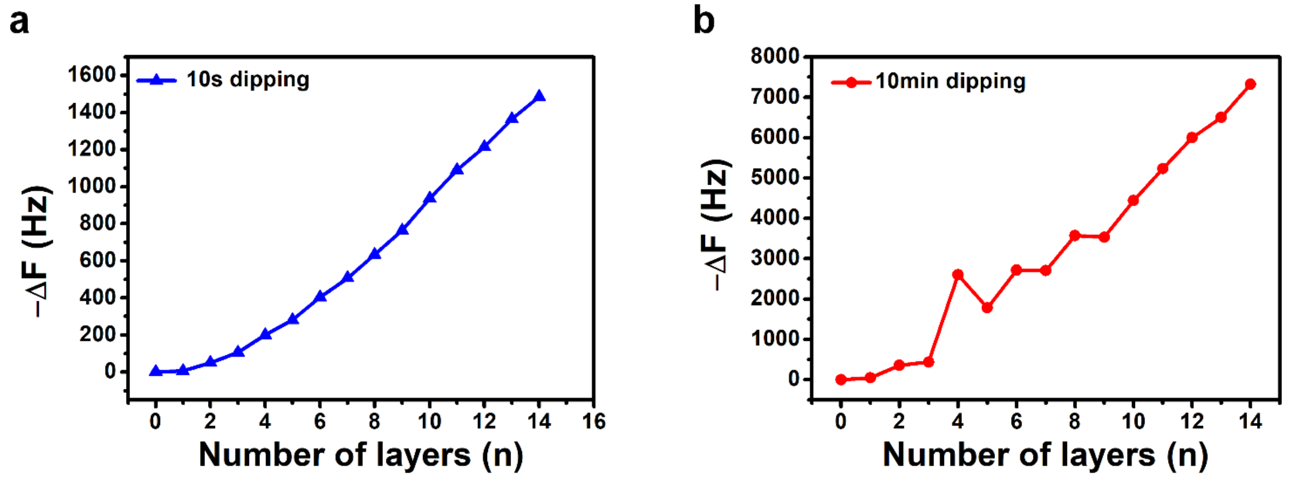

Figure S2 QCM analysis of the multilayer film up to 14 layers fabricated by the conventional dipping LbL method. (a) represents 10s and (b) represents 10min dipping time respectively.

Supplementary Figure S3

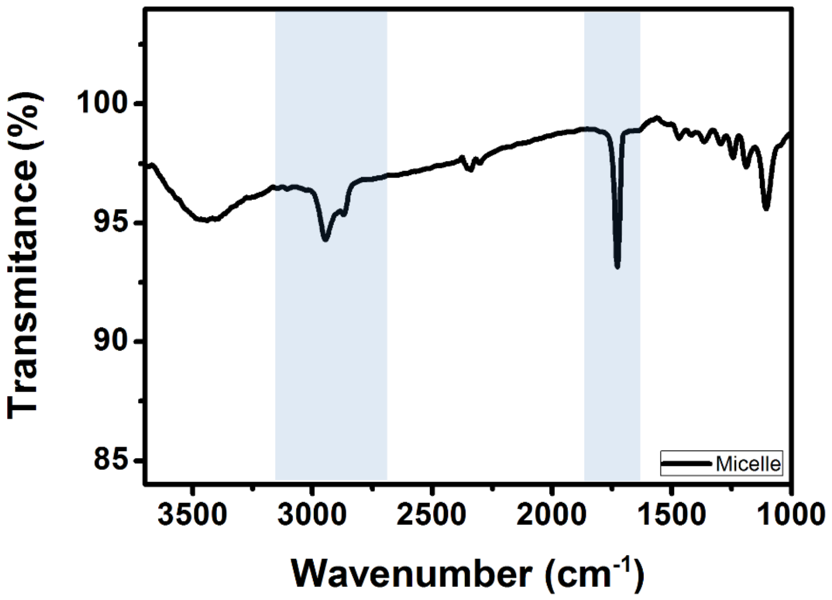

Figure S3 FT-IR spectrum of the dexamethasone-loaded PEO-*b*-PCL block copolymer micelles dispersed in deionized water without additional pH control.

Supplementary Figure S4

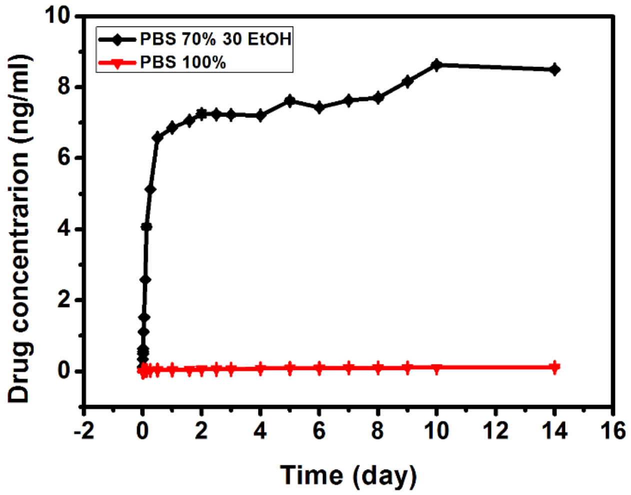

Figure S4 Comparison of the cumulative release profiles of coumarin 6, a model drug, in a mixture of PBS:EtOH (70:30 vol%) (black diamonds) and of 100% PBS (pH 7.4) at 37°C (red inverted tringles).
